# Optimization of HPAM Polymer Flooding for Enhanced Oil Recovery Through Experimental Core Flooding and Predictive Statistical Modeling

**DOI:** 10.3390/polym18131640

**Published:** 2026-07-01

**Authors:** Azizollah Khormali, Soroush Ahmadi

**Affiliations:** 1 Department of Chemistry, Faculty of Basic Sciences and Engineering, Gonbad Kavous University, Gonbad Kavous P.O. Box 4971799151, Iran; 2 Department of Chemical Engineering, Faculty of Petroleum, Gas, and Petrochemical Engineering, Persian Gulf University, Bushehr P.O. Box 7516913817, Iran

**Keywords:** HPAM polymer flooding, enhanced oil recovery, high salinity, response surface methodology, carbonate reservoirs

## Abstract

Polymer flooding is one of the most widely implemented chemical-enhanced oil recovery (EOR) techniques for improving sweep efficiency and mobilizing residual oil in mature reservoirs. However, the performance of partially hydrolyzed polyacrylamide (HPAM) flooding is strongly influenced by reservoir temperature, formation water salinity, and polymer concentration, particularly in carbonate formations where harsh reservoir conditions may significantly reduce polymer effectiveness. In this study, laboratory core flooding experiments combined with Response Surface Methodology (RSM) and Analysis of Variance (ANOVA) were employed to systematically investigate and optimize the effects of temperature, HPAM concentration, and salinity on the incremental recovery factor (RF) of matrix-type carbonate core samples. A total of 45 flooding experiments were conducted under temperatures ranging from 20 to 80 °C, polymer concentrations between 500 and 2500 ppm, and salinities from 1000 to 100,000 ppm. A highly significant quadratic model was developed, exhibiting excellent predictive capability (R^2^ = 0.9991, *p* < 0.0001) and accurately describing the individual and interactive effects of the investigated variables. Among the examined parameters, HPAM concentration was identified as the dominant factor controlling flooding performance, followed by salinity and temperature. The incremental recovery factor varied from approximately 6 to 19%, and optimization analysis predicted a maximum RF of 18.82% at 20 °C, 2500 ppm HPAM concentration, and 10,000 ppm salinity. Furthermore, optimization under high-temperature and high-salinity conditions revealed that a minimum HPAM concentration of about 2150 ppm is required to maintain RF values above 10%. The proposed experimental–statistical framework provides a reliable tool for predicting and optimizing HPAM flooding performance and offers practical guidance for polymer flooding design in carbonate reservoirs.

## 1. Introduction

The continuous growth in global energy demand has intensified the need for advanced technologies capable of maximizing oil recovery from existing reservoirs [1,2]. Although primary and secondary oil recovery methods have historically contributed significantly to hydrocarbon production, these conventional approaches typically recover only 20–40% of the original oil in place [3,4,5,6]. Water flooding remains the most common secondary recovery process in oilfield development because of its operational simplicity and relatively low cost [6,7,8,9]. However, one of the major limitations of conventional water flooding is its poor mobility control, particularly in heterogeneous reservoirs where injected water tends to preferentially flow through high-permeability channels, bypassing significant portions of oil in lower-permeability zones [3,4,10].

As a result, EOR technologies have become essential tools for extending reservoir life, improving production efficiency, and increasing the economic viability of mature oil fields [11,12,13]. Chemical-enhanced oil recovery has gained considerable attention because of its relatively broad applicability and effectiveness in improving displacement efficiency under a wide range of reservoir conditions [14,15,16]. Polymer flooding, one of the most established chemical EOR techniques, has emerged as a commercially successful and technically attractive strategy for improving oil recovery from both sandstone and carbonate reservoirs [17,18]. The primary mechanism of polymer flooding is based on modifying the mobility ratio between injected water and displaced oil through viscosity enhancement of the aqueous phase, thereby improving sweep efficiency and reducing viscous fingering and early water breakthrough [19,20].

Among various polymers investigated for EOR applications, partially hydrolyzed polyacrylamide (HPAM) is by far the most widely used and commercially implemented polymer. HPAM has gained dominance in field applications due to several advantages, including its strong viscosifying capability, relatively low cost, high molecular weight, availability, and extensive field validation [21,22]. Its ability to significantly increase aqueous phase viscosity at relatively low concentrations makes it particularly suitable for large-scale polymer flooding operations [23,24]. Despite its widespread application, the performance of HPAM is highly sensitive to reservoir and operational conditions [25,26]. The physicochemical behavior of HPAM in porous media is influenced by several factors, including temperature, salinity, pH, polymer concentration, molecular weight, shear rate, rock mineralogy, and the presence of divalent ions such as calcium and magnesium [27,28]. Consequently, identifying optimal operating conditions is critical for maximizing the effectiveness of polymer flooding while minimizing chemical cost and operational risks [29,30].

Among these influencing factors, temperature is one of the most critical variables affecting polymer flooding performance. Reservoir temperature directly influences polymer chain stability, viscosity retention, and long-term thermal degradation [22,31]. At elevated temperatures, HPAM molecules may undergo hydrolysis, chain scission, and oxidative degradation, leading to molecular weight reduction and loss of viscosifying power [31,32]. Another major operational factor is salinity, which significantly affects polymer solution behavior. Reservoir brines often contain high concentrations of dissolved salts, including monovalent ions such as sodium and potassium and divalent ions such as calcium and magnesium. These ions interact with negatively charged functional groups on HPAM molecules, causing contraction of the polymer molecular coil, reduced hydrodynamic volume, and lower solution viscosity [31,33]. The presence of high salinity can therefore diminish the effectiveness of HPAM flooding, especially in carbonate reservoirs where formation waters are often highly saline [34,35]. The third major parameter is polymer concentration, which directly determines the rheological behavior of the injected solution [36]. Increasing polymer concentration generally leads to higher solution viscosity and improved mobility control, resulting in better sweep efficiency and higher oil recovery [37,38]. However, excessively high polymer concentrations may create operational challenges such as injectivity reduction, increased pressure drop, pore plugging, and elevated project cost. The complexity of polymer flooding becomes even more significant when considering interaction effects among operational parameters. For example, the influence of polymer concentration may vary depending on reservoir temperature, while salinity effects may become more pronounced at elevated temperatures. Similarly, the optimum polymer dosage under low-salinity conditions may not remain effective in high-salinity reservoirs [39,40,41].

To overcome these limitations, advanced statistical and mathematical optimization tools have increasingly been adopted in petroleum engineering research. Among these, RSM has become one of the most widely used techniques for experimental design, process optimization, and predictive modeling [42,43,44]. RSM enables simultaneous evaluation of multiple independent variables and their interactions while minimizing the number of required experiments. By fitting a second-order polynomial model to experimental data, RSM allows prediction of system behavior across the design space and identification of optimum operating conditions [45,46]. One of the most powerful experimental designs used within RSM is Central Composite Design (CCD). CCD provides efficient estimation of linear, quadratic, and interaction effects while maintaining statistical robustness [46,47]. It is particularly suitable for chemical EOR studies where nonlinear relationships between operating variables and oil recovery are common. Compared with full factorial designs, CCD significantly reduces the experimental burden while preserving predictive capability [47]. Moreover, the reliability of RSM models is typically assessed using ANOVA, which quantifies the statistical significance of individual model terms and evaluates model adequacy through indicators such as *p*-values, F-values, coefficient of determination (R^2^), adjusted R^2^, predicted R^2^, and lack-of-fit tests. ANOVA enables researchers to distinguish statistically meaningful effects from experimental noise and provides confidence in optimization outcomes [48,49].

As polymer flooding performance is controlled by multiple interacting reservoir and operational parameters, researchers have increasingly adopted statistical optimization to identify the most influential factors and determine optimum operating conditions. In addition to viscosity enhancement and mobility control, the viscoelastic properties of HPAM may also contribute to improved microscopic displacement efficiency. Recently, Zhong et al. [50] investigated the interaction between polymer elasticity and wettability using oil droplet simulations and reported that increasing polymer elasticity enhanced oil displacement efficiency from 65.61% to 69.06% under strongly water-wet conditions, whereas the displacement efficiency decreased from 15.53% to 11.55% under weakly oil-wet conditions. Their study further revealed that polymer elasticity alters the distribution and orientation of normal stresses acting on trapped oil droplets, thereby affecting oil deformation and mobilization. Considering that carbonate reservoirs frequently exhibit mixed- or weakly oil-wet characteristics, the reduction in recovery factor observed in the present study at elevated temperatures and salinities may not only be associated with viscosity loss and polymer coil contraction but also with a partial deterioration of polymer elasticity. These findings suggest that, in addition to mobility control, viscoelastic effects and rock wettability should be considered when evaluating and optimizing polymer flooding performance in carbonate reservoirs.

Previous studies have successfully applied these techniques to optimize polymer concentration, slug size, injection strategy, mobility control parameters, and interfacial properties. Representative studies and their major findings are summarized in Table 1. As shown, most previous investigations focused on numerical simulations, surfactant–polymer systems, or mobility control optimization, whereas the present study develops an RSM–ANOVA model based on laboratory-scale HPAM flooding experiments in carbonate core samples under varying temperature, salinity, and polymer concentration conditions.

Despite the considerable progress achieved in optimizing polymer flooding operations, most published studies have focused on injection rate, injection timing, slug size, mobility-control parameters, economic indicators, or surfactant-polymer systems. Comparatively limited attention has been devoted to the combined experimental evaluation of temperature, polymer concentration, and salinity, particularly HPAM flooding in carbonate reservoirs. These variables are especially important because temperature affects polymer stability and viscosity retention, salinity influences polymer chain configuration and solution rheology, and polymer concentration directly controls mobility ratio and sweep efficiency. Furthermore, significant interaction effects among these variables are expected, making them particularly suitable for investigation through RSM and ANOVA. Therefore, the present study applies a systematic RSM-based experimental design to evaluate the individual and combined effects of temperature, salinity, and HPAM concentration on incremental recovery factor during carbonate core flooding, with the objective of developing a statistically validated predictive model and identifying optimum operating conditions for enhanced oil recovery. The main performance indicator considered is incremental recovery factor after two pore volumes of HPAM injection, representing the additional oil recovered beyond conventional water flooding. By integrating laboratory flooding experiments with RSM-based statistical modeling and ANOVA validation, this study aims to establish a predictive framework capable of identifying optimal polymer flooding conditions. Thus, the novelty of this work lies in the combined use of experimental core flooding and statistical optimization to evaluate HPAM flooding performance under realistic operational conditions relevant to carbonate reservoirs. Unlike purely experimental studies that focus only on observed trends, or purely modeling studies lacking physical validation, the proposed integrated methodology provides both mechanistic understanding and predictive capability. This combination enhances the scientific value of the work and improves its applicability to real field operations. It should be noted that the outcomes of this study are expected to provide several important contributions. First, they will improve understanding of how temperature, salinity, and HPAM concentration individually and collectively influence polymer flooding efficiency. Second, the developed predictive model will enable rapid estimation of incremental oil recovery under different operating scenarios, reducing the need for extensive laboratory testing. Third, the identified optimum operating conditions can support field-scale design and decision-making in future polymer flooding projects. Finally, the study will contribute to the broader advancement of chemical EOR technologies aimed at sustainable and economically efficient hydrocarbon production.

## 2. Materials and Methods

This study was designed to experimentally evaluate the performance of HPAM polymer flooding for enhanced oil recovery under varying operational conditions and to statistically optimize the process using RSM (Design-Expert^®^ software—version 13.0.5.0). The experimental work focused on determining the incremental recovery factor obtained after polymer injection in carbonate core samples, while the statistical phase aimed to model the relationship between operating parameters and recovery efficiency and to identify the optimum operating conditions. The three primary independent variables selected for investigation were temperature, polymer concentration, and salinity, which were chosen because they are among the most influential parameters affecting polymer performance in porous media.

### 2.1. Materials

The polymer selected for this study was partially hydrolyzed polyacrylamide (HPAM), specifically Flopaam^TM^ 3630S (SNF Floerger, Andrézieux, France), which is one of the most widely utilized polymers for chemical-enhanced oil recovery applications. HPAM was selected because of its extensive field application, excellent water solubility, high molecular weight, and strong capability to enhance the viscosity of the injected aqueous phase at relatively low concentrations. The polymer was supplied in dry granular powder form and possessed an average molecular weight of approximately 20 million Daltons (2 × 10^7^ Da), with a degree of hydrolysis ranging from 25 to 30%, which is consistent with commercial HPAM products commonly employed in polymer flooding operations. Prior to solution preparation, the polymer was stored in sealed moisture-free containers at ambient laboratory conditions to minimize moisture uptake, degradation, and contamination.

Synthetic formation waters were prepared using analytical-grade salts dissolved in deionized water to reproduce representative carbonate reservoir brines and ensure experimental repeatability. Unlike simple NaCl solutions, the prepared brines contained monovalent and divalent ions, including Na^+^, K^+^, Ca^2+^, Mg^2+^, Cl^−^, and SO_4_^2−^, thereby providing a more realistic representation of formation water chemistry encountered in carbonate reservoirs. Three synthetic formation waters with total dissolved solids (TDS) of approximately 10,000, 55,000, and 100,000 ppm were prepared to simulate low-, medium-, and high-salinity reservoir environments, respectively. The detailed ionic compositions of the synthetic brines are presented in Table 2. The inclusion of divalent cations is particularly important because Ca^2+^ and Mg^2+^ ions can significantly affect HPAM solution properties by reducing electrostatic repulsion between polymer segments, promoting polymer coil contraction, and consequently decreasing solution viscosity and mobility control capability.

The crude oil used in this work was a dead Iranian crude oil sample with a density of approximately 0.86 g/cm^3^ and a viscosity of about 10 cP at ambient conditions. The same oil batch was utilized throughout the entire experimental campaign to eliminate possible variations arising from differences in oil composition or rheological behavior.

The carbonate core samples employed in this study were classified as matrix-type carbonate cores and were carefully selected from the same rock block to minimize the influence of porous medium variability on polymer flooding performance. Visual inspection and petrophysical characterization confirmed the absence of natural fractures, large vugs, or fracture–vug networks commonly observed in some carbonate reservoirs, indicating that fluid flow was primarily governed by the interconnected matrix pore system. The selected core plugs exhibited relatively uniform petrophysical properties, with porosity ranging from 18 to 20% (average 19 ± 1.2%) and permeability varying between 24 and 30 mD (average 27 ± 3 mD). The permeability variation was less than ±10% from the average value, corresponding to a coefficient of variation below 12%, which indicates limited heterogeneity among the tested samples. Since all core plugs originated from the same carbonate formation and possessed comparable pore structures and petrophysical characteristics, they were considered relatively homogeneous at the laboratory scale. Consequently, variations in recovery factor among experiments can be primarily attributed to changes in temperature, HPAM concentration, and salinity rather than differences in rock properties.

The use of matrix-type carbonate cores was intentionally selected to minimize uncertainties associated with fracture flow and complex dual-porosity behavior, thereby allowing a systematic evaluation of the effects of HPAM concentration, temperature, and salinity on polymer flooding performance. All core plugs were obtained from the same carbonate rock block and were selected to possess comparable pore structures and petrophysical properties. This approach ensured that variations in recovery factor were predominantly associated with changes in the investigated operational parameters rather than differences in rock characteristics. Although naturally fractured and vuggy carbonate reservoirs constitute an important class of hydrocarbon reservoirs, their flow mechanisms involve additional complexities such as fracture–matrix interaction, gravity drainage, and preferential flow channels. Investigation of polymer flooding under such conditions is beyond the scope of the present work and may be considered in future studies.

Porosity was determined using the gravimetric saturation method, while permeability was measured using a gas permeameter (Vinci technology, Nanterre, France). Each core plug was individually characterized before use to ensure consistency among experimental runs. Prior to flooding experiments, all carbonate core samples were cleaned to remove residual contaminants, oils, and moisture. Cleaning was performed using solvent extraction with toluene and methanol until the effluent appeared clear. The cleaned cores were then oven-dried at 105 °C for 24 h to remove residual solvents and water. After drying, each core was weighed and its dimensions were measured accurately to calculate bulk volume.

Wettability is another important parameter governing polymer flooding efficiency because it affects fluid distribution, capillary trapping, and residual oil saturation. The carbonate cores employed in this study were assumed to exhibit weakly oil-wet to intermediate-wet characteristics, which are commonly encountered in carbonate reservoirs. To establish comparable initial conditions, all cores were saturated with synthetic brine and subsequently flooded with crude oil following identical procedures to attain similar initial water saturation and oil saturation values. Water flooding was then continued until a produced water cut exceeding 95% was achieved, ensuring that residual oil saturation prior to polymer injection was nearly identical among all experiments.

Consequently, porous medium characteristics, wettability conditions, and crude oil properties were intentionally maintained nearly constant, allowing the present study to specifically quantify the effects of temperature, HPAM concentration, and salinity on the incremental recovery factor obtained by polymer flooding.

### 2.2. Preparation of Polymer Solutions

Polymer solutions were prepared freshly before each experimental run to minimize degradation and ensure consistency. The required mass of HPAM for each target concentration was calculated based on the desired solution volume using the standard concentration relationship:

(1) $$m=C\times V\times 10^{-6}$$

where *m* is the mass of polymer (g), *C* is the desired polymer concentration (ppm), and *V* is the solution volume (mL).

The prepared synthetic brine was first transferred into a clean mixing vessel. HPAM powder was then added gradually and uniformly to the brine while stirring with a magnetic stirrer (Heidolph, Schwabach, Germany) at approximately 500 rpm. This stirring speed was selected because it provides sufficient mixing energy to facilitate dissolution and prevent agglomeration and fish-eye formation, while remaining low enough to minimize shear-induced degradation and chain scission of the high-molecular-weight HPAM molecules. The mixture was stirred continuously for 4 h, which was found to be adequate for complete dissolution of the polymer particles. Subsequently, the prepared solution was allowed to hydrate undisturbed at room temperature for 24 h. This hydration period was employed to ensure complete swelling and molecular expansion of the polymer chains and to allow the solution viscosity to reach equilibrium conditions. Prior to injection, each polymer solution was visually inspected to confirm the absence of undissolved particles or gel-like agglomerates, indicating successful preparation of a homogeneous polymer solution.

Table 3 presents the variation in HPAM solution viscosity with polymer concentration at a constant temperature of 25 °C and a salinity of 55,000 ppm. The results indicate a significant increase in viscosity as polymer concentration increases. Specifically, increasing the HPAM concentration from 500 to 2500 ppm enhanced the solution viscosity from approximately 4.5 cP to 24.0 cP, corresponding to a more than fivefold increase. This behavior can be attributed to the higher degree of intermolecular interactions, polymer chain overlap, and entanglement occurring at elevated concentrations, which increases the hydrodynamic volume of the polymer molecules. The substantial viscosity enhancement contributes to improved mobility control and sweep efficiency during polymer flooding.

Table 4 summarizes the influence of temperature on the viscosity of a 1500 ppm HPAM solution prepared in synthetic formation water with a salinity of 55,000 ppm. As expected, viscosity decreases progressively with increasing temperature, declining from 13.5 cP at 20 °C to 7.2 cP at 80 °C, which corresponds to an overall reduction of approximately 47%. This reduction is mainly associated with enhanced thermal motion of polymer chains, weakening of intermolecular associations, and partial thermal degradation at elevated temperatures. The observed decrease in viscosity explains, at least partially, the lower recovery factors obtained during polymer flooding experiments conducted under high-temperature conditions.

### 2.3. Experimental Procedure

(I) Before polymer injection, a conventional water flooding stage was conducted to simulate secondary oil recovery. Synthetic brine was injected into each oil-saturated core at a constant flow rate until water breakthrough occurred and the produced water cut exceeded 95%. At this stage, the remaining oil represented residual oil saturation after water flooding, which served as the baseline condition for polymer flooding. This step was essential because the purpose of the study was to evaluate the incremental oil recovery attributable specifically to HPAM flooding beyond traditional water flooding performance.

(II) Polymer flooding experiments were performed using a laboratory-scale core flooding system (Vinci technology, Nanterre, France) consisting of a high-pressure injection pump, stainless steel core holder, temperature-controlled oven, pressure transducers, and effluent collection units.

(III) Each saturated core was placed inside the core holder and brought to the desired experimental temperature (20, 50, or 80 °C). The prepared HPAM solution at the selected concentration and salinity was then injected into the core at a constant flow rate. A polymer slug volume equivalent to two pore volumes (2 PV) was injected in each experiment. Although field-scale polymer flooding operations typically employ polymer slug sizes ranging from approximately 0.3 to 0.5 PV to balance recovery improvement and chemical cost, larger injection volumes are frequently adopted in laboratory core flooding studies to evaluate the maximum displacement capability of polymer systems and to ensure that the recovery response reaches a near-stabilized condition. The purpose of the present study was to investigate the influence of temperature, HPAM concentration, and salinity on the incremental recovery factor rather than to optimize polymer slug size. Therefore, a constant injection volume of 2 PV was selected for all experiments to provide sufficient interaction between the injected polymer solution and the residual oil phase and to facilitate reliable comparison of flooding performance under different operating conditions. Beyond approximately 2 PV, additional oil production was observed to be negligible, indicating that the majority of recoverable oil mobilized by polymer flooding had already been displaced.

(IV) Produced fluids were collected continuously throughout the flooding process. The produced oil volume was measured using a calibrated separator (Duran, Schott, Mainz, Germany), and pressure drop across the core was monitored during injection to detect possible injectivity issues or plugging effects.

(V) The primary response variable used in this study was the incremental recovery factor (RF), which represents the additional oil recovered during polymer flooding relative to the original oil in place (OOIP). It was calculated using:

(2) $$RF\%=\frac{V_{o-p}}{OOIP}\times 100$$

where V_o−p_ is the volume of oil recovered during polymer flooding (mL). This metric was selected because it directly quantifies the effectiveness of polymer flooding and is commonly used in enhanced oil recovery studies.

### 2.4. RSM

RSM is a powerful statistical and mathematical technique that combines experimental design, regression analysis, and optimization procedures to evaluate the relationships between multiple independent variables and one or more response variables. Compared with conventional one-factor-at-a-time approaches, RSM significantly reduces the number of required experiments while simultaneously identifying individual effects, interaction effects, and quadratic influences of the investigated parameters [52,53,55]. In addition, RSM provides a reliable predictive model that can be used to estimate system performance within the experimental domain and determine optimum operating conditions. In the present study, RSM was employed to investigate the influence of three important operational parameters affecting HPAM polymer flooding performance in carbonate reservoirs, namely temperature (A), HPAM concentration (B), and salinity (C). These parameters were selected because they strongly influence polymer rheology, thermal stability, molecular configuration, mobility control, and ultimately oil recovery efficiency. Temperature was varied between 20 and 80 °C, HPAM concentration between 500 and 2500 ppm, and salinity between 10,000 and 100,000 ppm. The selected ranges were chosen to represent typical conditions encountered in low- to moderately high-temperature carbonate reservoirs and to adequately cover the practical operating window of HPAM flooding processes. To facilitate statistical analysis and model development, the independent variables were transformed into coded values. The coded levels of −1, 0, and +1 represent the low, center, and high levels of each parameter, respectively. The actual and coded values of the investigated parameters are presented in Table 5.

The investigated parameter ranges were selected based on practical considerations and previously reported operating conditions for HPAM flooding applications. The temperature range of 20–80 °C represents reservoir environments varying from shallow formations to moderately high-temperature carbonate reservoirs. HPAM concentration was varied between 500 and 2500 ppm because this interval generally provides sufficient viscosity enhancement while maintaining acceptable injectivity and chemical consumption. Concentrations higher than approximately 2500 ppm may considerably increase injection pressure and operational costs. In addition, the salinity range of 10,000–100,000 ppm was intentionally chosen to encompass both favorable and highly challenging reservoir conditions. It is well recognized that conventional partially hydrolyzed polyacrylamide exhibits limited tolerance toward elevated salinity due to charge screening effects caused by dissolved ions. Increasing ionic strength reduces electrostatic repulsion between polymer segments, leading to contraction of polymer coils, a decrease in hydrodynamic radius, and consequently a significant reduction in solution viscosity. At very high salinity levels, polymer aggregation or precipitation may occur depending on the ionic composition of the brine, particularly in the presence of divalent cations. Nevertheless, the prepared HPAM solutions employed in this study remained visually homogeneous throughout solution preparation and flooding experiments. Therefore, the salinity level of 100,000 ppm was considered as an upper-bound condition to evaluate the applicability limits of conventional HPAM flooding and to identify polymer concentrations capable of sustaining acceptable oil recovery under severe reservoir environments.

A design of experiments (DOE) matrix was subsequently developed to systematically explore the experimental domain. A total of 45 experimental runs were conducted, corresponding to different combinations of temperature, HPAM concentration, and salinity. The selected number of runs ensured sufficient coverage of the design space and allowed accurate estimation of linear, quadratic, and interaction effects among the variables. Each experimental run consisted of a polymer flooding test performed under the specified operating conditions, and the resulting incremental recovery factor was recorded as the response variable. The complete experimental matrix together with the measured responses is presented in Table 6. The experimental results were analyzed using a second-order polynomial model, which is commonly employed in RSM studies because of its ability to describe both linear and nonlinear system behavior. The general form of the developed regression model is expressed as:

(3) $$Y=\beta_{0}+\sum \beta_{i}X_{i}+\sum \beta_{ii}X_{i}^{2}+\sum \sum \beta_{ii}X_{i}X_{j}\;\;(i\;and\;j=1,\;2,\;3,\;\ldots \;K)$$

where Y represents the predicted response (recovery factor), β_0_ is the intercept coefficient, β_i_ represents the linear coefficients, β_ii_ denotes the quadratic coefficients, and β_ij_ corresponds to the interaction coefficients between variables X_i_ and X_j_. The terms X_i_ and X_j_ represent the independent variables.

**Figure 1:**
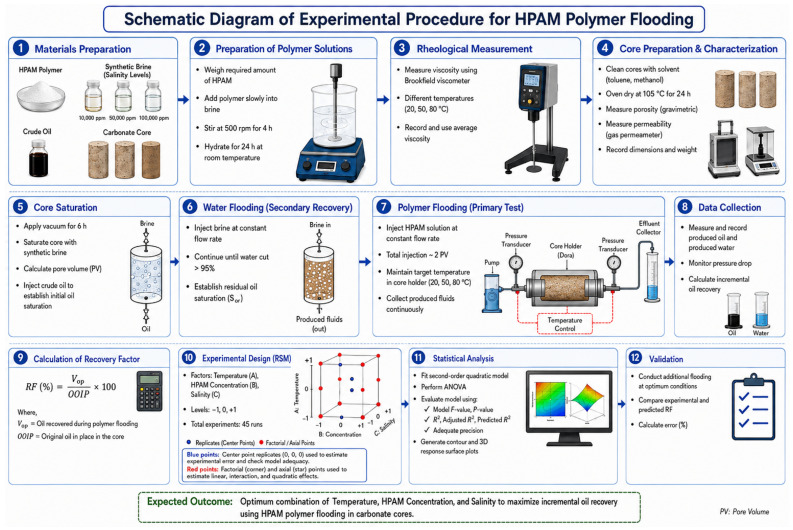
Schematic representation of the HPAM flooding experimental and modeling procedure.

Figure 1 presents a schematic overview of the experimental and statistical methodology employed in this study to evaluate the performance of HPAM polymer flooding in carbonate core samples. The workflow begins with the preparation of experimental materials, including HPAM polymer, synthetic brine solutions with different salinity levels, crude oil, and carbonate core plugs. Polymer solutions were subsequently prepared at various concentrations and characterized through rheological measurements to determine their viscosity behavior under different temperatures. The carbonate cores were cleaned, characterized, saturated with brine and crude oil, and subjected to conventional water flooding to establish residual oil saturation conditions. Polymer flooding experiments were then performed by injecting HPAM solutions under different temperature, concentration, and salinity conditions up to 2 pore volumes. The produced fluids were collected and used to calculate the incremental recovery factor. Finally, the experimental data were analyzed using Response Surface Methodology to develop predictive models, evaluate parameter interactions, determine statistically significant factors, and identify the optimum operating conditions for maximizing oil recovery.

## 3. Results and Discussion

### 3.1. Analysis of Variance (ANOVA)

ANOVA was employed to evaluate the statistical significance, adequacy, and reliability of the RSM model for predicting the recovery factor during HPAM polymer flooding. ANOVA partitions the total variation in the response into contributions from model terms and experimental error, enabling identification of significant factors and interactions [56]. In general, the significance of a model or individual term is assessed through the F-value and *p*-value. A high F-value indicates that the corresponding model term has a strong influence on the response, whereas a low *p*-value indicates that the observed effect is statistically significant and unlikely to be caused by random experimental variations [57]. At a confidence level of 95%, model terms with *p*-values less than 0.05 are considered statistically significant.

The ANOVA results for the recovery factor are summarized in Table 7. The results demonstrate that the developed quadratic model is highly significant, as evidenced by its extremely low *p*-value (*p* < 0.0001). This confirms that the relationship between temperature, HPAM concentration, salinity, and recovery factor is statistically meaningful and can be accurately represented by the proposed regression equation. Furthermore, the model exhibits a very high F-value (5229.02), indicating strong model significance and a negligible probability of noise-induced effects. Consequently, the developed model can be considered highly reliable for describing the behavior of the investigated system within the selected experimental domain.

A detailed examination of the ANOVA results reveals that all three primary variables, temperature (A), HPAM concentration (B), and salinity (C), significantly influence the recovery factor. In addition, the interaction terms AB, AC, and BC are statistically significant, indicating that the effect of one parameter depends on the level of another parameter. Such interactions are expected in polymer flooding systems because polymer rheology, thermal stability, and ionic sensitivity are strongly interrelated. The significance of these interaction terms confirms the necessity of employing multivariable optimization techniques such as RSM rather than conventional one-factor-at-a-time approaches.

The quadratic effect of polymer concentration (B^2^) was also found to be significant, indicating the existence of nonlinear behavior within the investigated concentration range. This observation suggests that increasing polymer concentration does not produce a strictly linear increase in recovery factor and that an optimum concentration region exists. The significance of the quadratic term is consistent with the physical behavior of polymer solutions, where viscosity enhancement and mobility control improve with concentration but eventually exhibit diminishing returns due to molecular entanglement and injectivity limitations. In contrast, the quadratic effect of salinity (C^2^) showed a comparatively smaller contribution to the model, although it remained important for accurately describing the response surface.

Based on F-values, HPAM concentration emerges as the most influential parameter, followed by salinity and temperature. This result is consistent with the fundamental mechanisms of polymer flooding. Polymer concentration directly controls solution viscosity and mobility ratio, thereby exerting a dominant influence on sweep efficiency. Salinity ranks second because dissolved ions significantly affect polymer chain expansion, hydrodynamic volume, and viscosity retention. Temperature exhibits the lowest contribution among the three main factors, although its influence remains statistically significant due to its effect on polymer degradation and viscosity reduction. Based on the ANOVA results, the relative importance of the investigated parameters can be ranked as follows: B > C > A > BC > AB > AC > B^2^ > C^2^.

ANOVA results demonstrated that HPAM concentration is the dominant parameter governing polymer flooding performance, followed by salinity and temperature. The relative importance of the investigated variables was determined to be HPAM concentration > salinity > temperature. Increasing polymer concentration substantially improves solution viscosity and mobility control, leading to higher sweep efficiency and incremental oil recovery. In contrast, increasing salinity compresses polymer chains due to electrostatic shielding effects, while increasing temperature decreases solution viscosity and may induce partial thermal degradation of HPAM molecules. Consequently, although both temperature and salinity adversely affect polymer flooding efficiency, their influence is less pronounced than that of polymer concentration within the investigated experimental range.

This ranking clearly demonstrates that optimizing polymer concentration is the most effective strategy for maximizing recovery factor, while salinity control and temperature management also play important roles in improving HPAM flooding performance.

### 3.2. Fit-Statistics

The adequacy and predictive capability of the developed quadratic model were further assessed using several statistical indicators, including the coefficient of determination (R^2^), adjusted coefficient of determination (Adj-R^2^), predicted coefficient of determination (Pred-R^2^), coefficient of variation (C.V.), and adequate precision (A.P.). These parameters are commonly used to evaluate the quality of RSM models and to verify whether the developed regression equation can accurately describe and predict experimental observations.

The statistical parameters presented in Table 8 indicate excellent model performance. The coefficient of determination (R^2^) was calculated as 0.9991, indicating that approximately 99.91% of the variation in recovery factor can be explained by the developed regression model. Such a high R^2^ value confirms the excellent agreement between the experimental data and model predictions and suggests that only a very small fraction of the observed variation remains unexplained [58,59].

While R^2^ alone may overestimate model performance, adjusted and predicted R^2^ provide a more robust evaluation of model reliability. The Adj-R^2^ and Pred-R^2^ values were determined as 0.9989 and 0.9986, respectively. The difference between these values is less than 0.003, which is considerably below the generally accepted threshold of 0.20. The close agreement among R^2^, Adj-R^2^, and Pred-R^2^ confirms that the model possesses excellent predictive capability and is free from overfitting. This observation indicates that the developed model can reliably predict recovery factor values not only for the experimental points used during model development but also for new operating conditions within the investigated design space.

Another important indicator of model quality is the adequate precision, which measures the signal-to-noise ratio of the model. A value greater than 4 is generally considered desirable for a robust predictive model [60]. In the present study, the adequate precision was found to be 288.7618, which is exceptionally high and far exceeds the minimum requirement. This result demonstrates that the model provides a very strong signal relative to the experimental noise and can effectively navigate the design space for optimization purposes.

The coefficient of variation represents the ratio of the standard deviation to the mean response and is used as a measure of experimental reproducibility and precision [61]. The obtained C.V. value of 1.45% is remarkably low, indicating excellent repeatability of the experimental measurements and minimal random error. Generally, C.V. values below 10% are considered acceptable for experimental studies, while values below 5% indicate highly reliable experimental data. Therefore, the low C.V. obtained in this study further confirms the quality of the experimental procedure and the robustness of the resulting model.

Collectively, the ANOVA results and fit statistics demonstrate that the developed quadratic model provides an accurate, reliable, and statistically significant representation of the HPAM flooding process. The strong agreement between experimental and predicted values, together with the high R^2^ values, low coefficient of variation, and exceptionally large adequate precision, confirms the suitability of the model for evaluating parameter effects, generating response surfaces, and determining optimum operating conditions for maximizing the recovery factor. Based on these findings, the reduced quadratic regression equation presented below was selected as the final predictive model for recovery factor in terms of coded variables.

***RF*^1.6^** = 48.1861 − 9.8743*A* + 24.6633*B* − 12.1044*C* − 4.27537*AB* + 2.05663*AC* − 5.14142*BC* + 2.86241*B*^2^ + 0.378791*C*^2^ (4)

### 3.3. Model Adequacy Testing and Validation (Diagnostics Plots)

The adequacy, reliability, and predictive capability of the developed RSM model were further evaluated through a series of diagnostic plots. Diagnostic analysis is essential for validating regression assumptions and evaluating model suitability for prediction and optimization. The diagnostic plots examined in this study include the normal probability plot of residuals, residuals versus predicted values, residuals versus run order, and predicted versus actual values. Collectively, these plots provide valuable information regarding residual normality, homoscedasticity (constant variance), independence of errors, and overall model accuracy.

**Figure 2:**
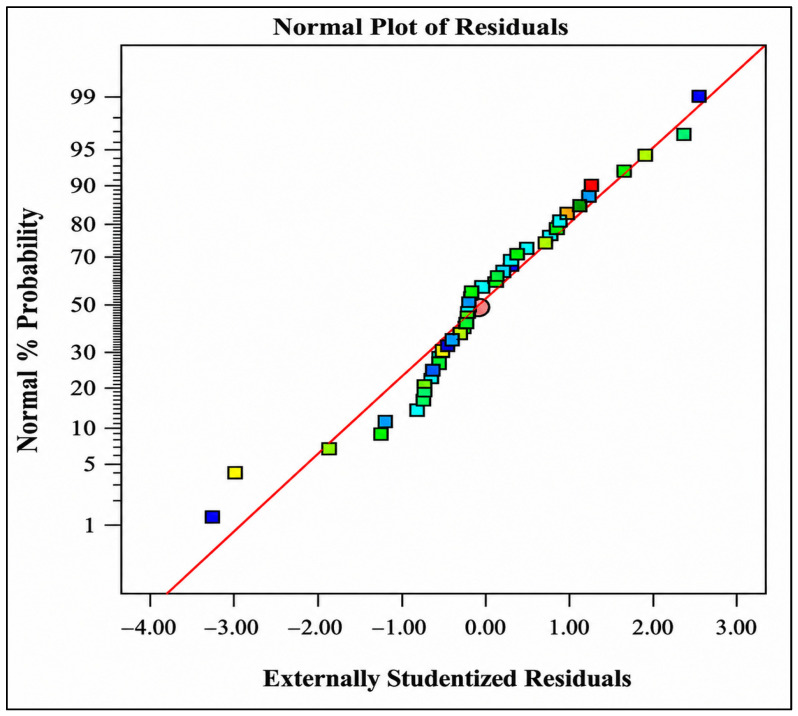
Normal probability plot of externally studentized residuals for the developed RF model.

Figure 2 presents the normal probability plot of externally studentized residuals for the developed recovery factor model. This plot is commonly used to assess whether the residuals follow a normal distribution, which is one of the fundamental assumptions of regression modeling and ANOVA. In an adequate model, the residual points should lie approximately along a straight diagonal line [62]. As observed in Figure 2, the majority of the residuals closely follow the reference line with only minor deviations at the extreme ends of the distribution. The absence of significant departures from linearity indicates that the residuals are normally distributed and that no major transformation of the response variable is required. Furthermore, the relatively symmetrical distribution of residuals around the center of the plot suggests that the model errors are random rather than systematic [63]. Therefore, the normality assumption required for ANOVA and regression analysis is satisfactorily fulfilled.

**Figure 3:**
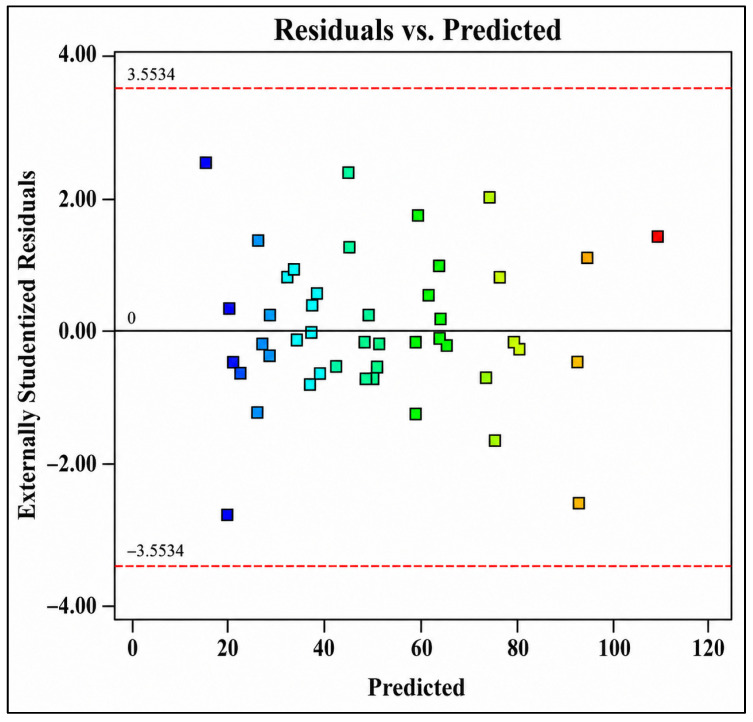
Residuals versus predicted values for the developed RF model.

The residuals versus predicted values plot for the RF model is illustrated in Figure 3. This diagnostic graph is used to evaluate whether the residuals exhibit constant variance throughout the prediction range and to identify potential model deficiencies such as nonlinearity, heteroscedasticity, or missing terms. Ideally, the residuals should be randomly scattered around the zero line without forming any distinct pattern [64]. As shown in Figure 3, the residual points are distributed randomly on both sides of the centerline and remain within the externally studentized residual limits of ±3.5534. No funnel-shaped distribution, curvature, clustering, or systematic trend is observed. The relatively uniform scatter of points across the entire prediction range demonstrates that the variance remains essentially constant and that the model adequately captures the relationship between the independent variables and recovery factor. The absence of outliers beyond the confidence limits further confirms the robustness of the developed regression model.

**Figure 4:**
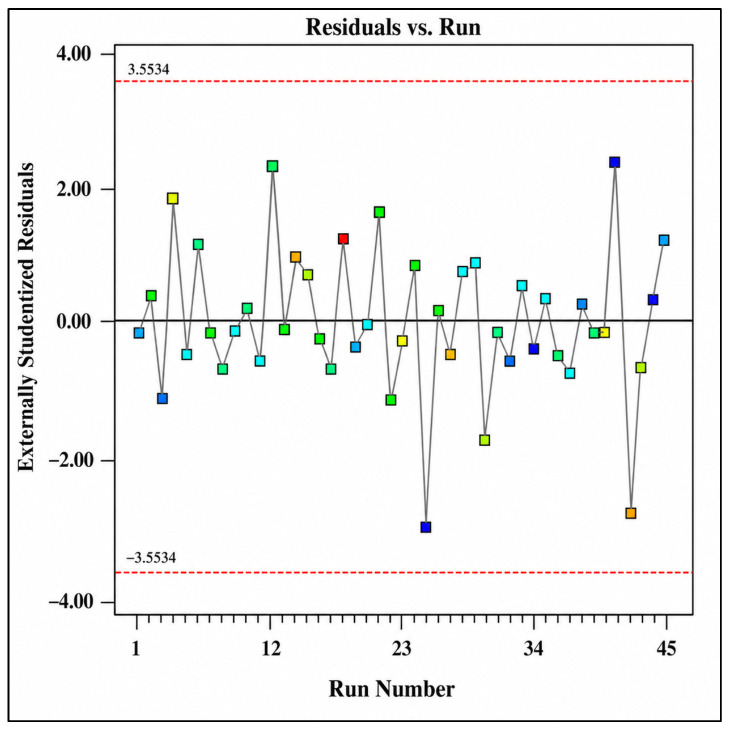
Residuals versus run order for the developed RF model.

Figure 4 depicts the residuals plotted against run order. This graph is particularly useful for examining the independence of experimental errors and detecting potential time-related effects such as instrument drift, environmental changes, operator bias, or systematic experimental errors. For a statistically sound model, the residuals should fluctuate randomly around the zero line without displaying any increasing, decreasing, cyclic, or repeating pattern [65]. Examination of Figure 4 reveals that the residuals are randomly distributed throughout the experimental sequence and remain within the acceptable studentized residual limits. No noticeable trend or serial correlation is observed among consecutive runs. The random dispersion of residuals confirms that the experimental data were collected under stable conditions and that the observed variations in recovery factor are primarily attributable to the investigated factors rather than uncontrolled experimental influences. Consequently, the assumption of residual independence is satisfied.

**Figure 5:**
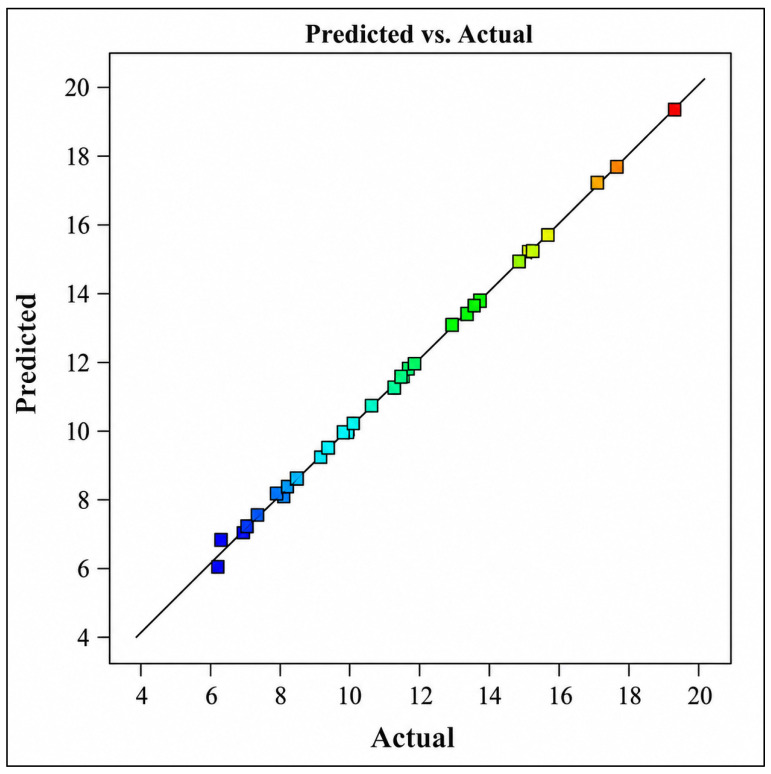
Comparison of predicted and experimental recovery factor values.

The predictive performance of the developed model was further assessed using the predicted versus actual values plot shown in Figure 5. This plot provides a direct comparison between experimentally measured recovery factors and those predicted by the developed quadratic model. Ideally, all data points should lie on or very close to the diagonal line, indicating perfect agreement between measured and predicted values [66]. As illustrated in Figure 5, virtually all experimental points are located directly on the diagonal line with negligible deviation. The exceptional agreement between predicted and actual values confirms the high accuracy of the model and is consistent with the extremely high coefficient of determination (R^2^ = 0.9991) obtained from the ANOVA analysis. The close correspondence between measured and predicted responses demonstrates that the developed model successfully captures the influence of temperature, HPAM concentration, and salinity on the recovery factor and possesses excellent predictive capability within the investigated experimental domain.

Consequently, the diagnostic analyses presented in Figure 2, Figure 3, Figure 4 and Figure 5 provide strong evidence of the validity and adequacy of the developed RF model. The normal probability plot confirms the normal distribution of residuals; the residuals versus predicted plot verifies constant variance; the residuals versus run order plot demonstrates independence of errors; and the predicted versus actual plot confirms excellent predictive accuracy. The absence of significant outliers, systematic trends, or model deficiencies indicates that the assumptions required for ANOVA and regression analysis are fully satisfied. Therefore, the developed RSM model can be considered statistically reliable and suitable for evaluating parameter effects, generating response surfaces, and optimizing HPAM polymer flooding performance in carbonate reservoirs.

### 3.4. Influence of Operating Parameters

**Figure 6:**
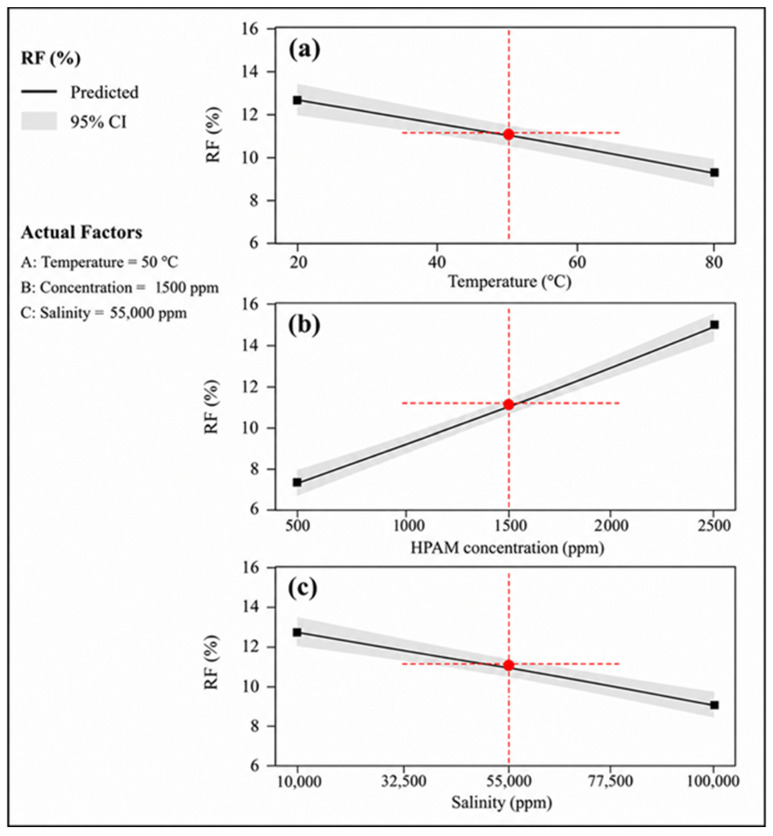
The effect of parameters on the recovery factor, temperature effect (**a**); HPAM concentration effect (**b**); and salinity effect (**c**).

**Figure 7:**
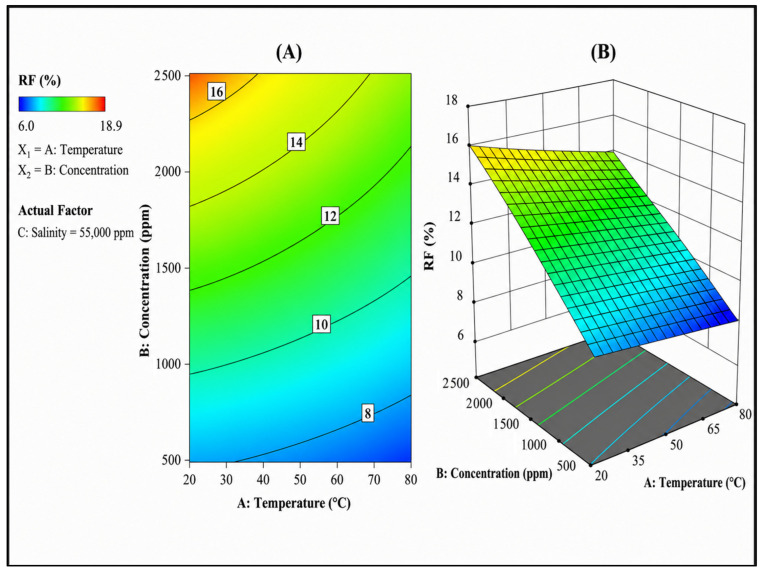
Interaction effect of the temperature and HPAM concentration on the recovery factor, contour plot (**A**) and 3-D plot (**B**).

**Figure 8:**
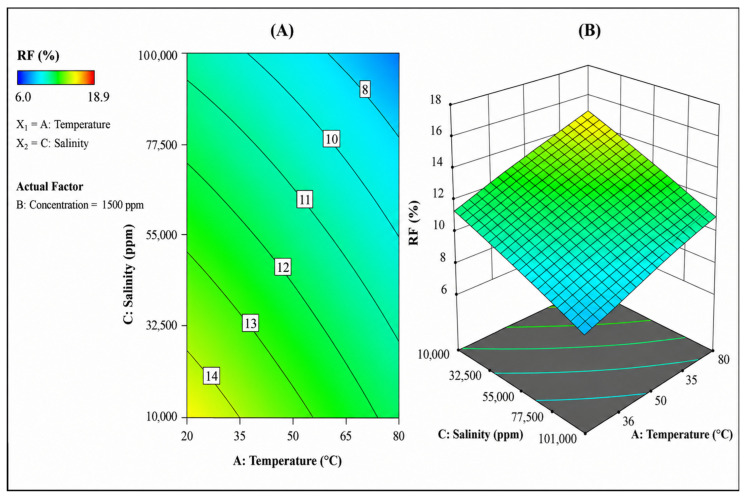
Interaction effect of the temperature and salinity on the recovery factor, contour plot (**A**) and 3-D plot (**B**).

**Figure 9:**
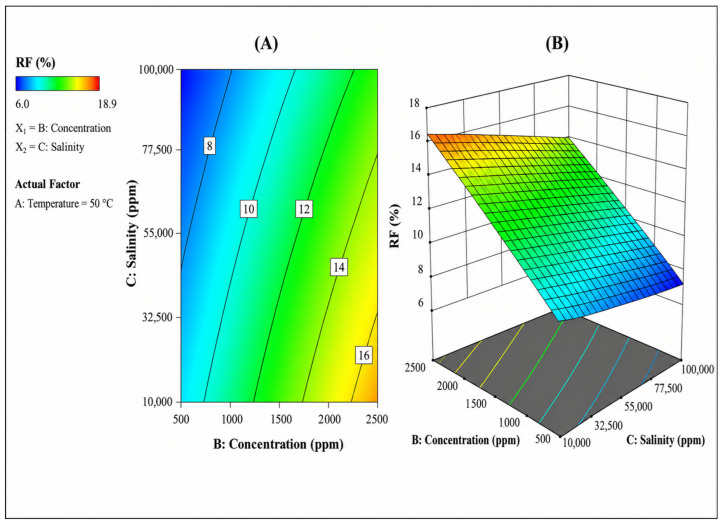
Interaction effect of the salinity and HPAM concentration on the recovery factor, contour plot (**A**) and 3-D plot (**B**).

The developed RSM model was utilized to evaluate the effects of temperature, HPAM concentration, and salinity on the recovery factor through one-factor response plots, contour plots, and three-dimensional response surface plots. These graphical analyses provide a clear visualization of both the individual and interactive effects of the investigated variables within the selected experimental domain. In the one-factor plots, the effect of a single parameter on RF is examined while the remaining variables are maintained at their center levels (coded value of 0), allowing direct assessment of the main effects. Similarly, contour and response surface plots illustrate the simultaneous influence of two variables on the recovery factor while the third variable is fixed at its central level. These plots are particularly useful for identifying parameter interactions, understanding the response behavior, and locating the optimum operating region [67]. The observed trends in Figure 6, Figure 7, Figure 8 and Figure 9 are consistent with the ANOVA results and confirm that temperature, HPAM concentration, and salinity significantly influence HPAM flooding performance in carbonate core samples.

Figure 6 presents the one-factor response plots generated from the RSM model, illustrating the individual influence of temperature, HPAM concentration, and salinity on the recovery factor. These plots provide valuable insight into the relative importance of each parameter and confirm the trends predicted by the ANOVA-derived regression model. Figure 6a shows the influence of temperature on the recovery factor while maintaining the HPAM concentration at 1500 ppm and salinity at 55,000 ppm. The recovery factor decreases almost linearly with increasing temperature. At approximately 20 °C, the predicted recovery factor is close to 12.5%, whereas at 80 °C it decreases to nearly 10%, representing a reduction of approximately 20%. This decline demonstrates the adverse impact of temperature on HPAM flooding efficiency. Elevated temperatures accelerate thermal degradation of HPAM molecules through hydrolysis, chain scission, and oxidative reactions [68]. As the polymer chains become shorter, their hydrodynamic volume decreases, resulting in lower solution viscosity. Since the primary mechanism of polymer flooding is mobility control through viscosity enhancement, any reduction in viscosity directly decreases sweep efficiency and consequently oil recovery [45]. Moreover, higher temperatures increase molecular motion and reduce intermolecular interactions within the polymer solution [69]. The contraction of polymer coils and reduction in viscoelastic properties diminish the ability of HPAM to improve microscopic displacement efficiency. The negative slope observed in Figure 6a confirms that temperature is a statistically significant factor affecting recovery, as also indicated by the ANOVA analysis. Figure 6b illustrates the effect of polymer concentration while temperature and salinity are fixed at 50 °C and 55,000 ppm, respectively. Unlike temperature, recovery factor increases significantly with increasing HPAM concentration. The RF rises from approximately 8% at 500 ppm to nearly 15% at 2500 ppm, corresponding to an increase of almost 87%. The positive effect of polymer concentration is attributed to the increase in solution viscosity. Higher polymer concentrations create stronger intermolecular entanglements and larger hydrodynamic volumes, thereby increasing resistance to flow [70]. This reduces the mobility ratio between injected water and reservoir oil and minimizes viscous fingering. Consequently, a larger portion of the reservoir is swept by the displacing fluid. The strong positive slope indicates that HPAM concentration is likely the most influential variable among the three investigated parameters. This observation agrees with polymer flooding theory, where polymer concentration directly controls mobility ratio and sweep efficiency. Figure 6c shows the effect of salinity while temperature and HPAM concentration are fixed at 50 °C and 1500 ppm, respectively. Increasing salinity from 10,000 ppm to 100,000 ppm causes the recovery factor to decrease from approximately 13% to around 9.5%, corresponding to a reduction of roughly 27%. This behavior is primarily related to the polyelectrolyte nature of HPAM. The negatively charged carboxylate groups present on polymer chains repel each other under low-salinity conditions, causing the polymer coils to expand. As salinity increases, dissolved ions shield these electrostatic repulsions, resulting in polymer coil contraction [71]. Consequently, the effective hydrodynamic volume decreases and solution viscosity is reduced. In addition, high salinity may promote polymer adsorption on rock surfaces and reduce injectivity performance. The decline observed in Figure 6c confirms the detrimental effect of saline environments on HPAM flooding efficiency and highlights the importance of salinity management in polymer EOR projects.

Figure 7 presents contour and three-dimensional response surface plots describing the combined effects of temperature and HPAM concentration on recovery factor while salinity is maintained constant at 55,000 ppm. Recovery factor values increase steadily from the lower-right region (high temperature and low concentration) toward the upper-left region (low temperature and high concentration). The lowest predicted recovery factor is approximately 7–8%, while the highest predicted value approaches 16–17%. The response surface further demonstrates that increasing HPAM concentration consistently enhances recovery across the entire temperature range. For example, at 20 °C, increasing concentration from 500 ppm to 2500 ppm raises RF from approximately 9% to nearly 17%, corresponding to an improvement of almost 89%. Similarly, at 80 °C, recovery increases from approximately 7% to about 13%. Temperature exerts the opposite influence. At a fixed concentration of 1500 ppm, increasing temperature from 20 °C to 80 °C decreases recovery from approximately 12.5% to around 10%. The relatively planar surface indicates that the interaction between temperature and concentration is moderate rather than highly nonlinear. Mechanistically, increasing polymer concentration enhances mobility control by increasing viscosity, whereas increasing temperature promotes polymer degradation and viscosity loss [72]. The contour orientation suggests that the beneficial effect of concentration partially compensates for temperature-induced performance losses. This finding is important because it implies that higher polymer dosages may be required in relatively hot reservoirs to maintain acceptable recovery performance.

Figure 8 presents the contour and three-dimensional response surface plots describing the interaction between temperature and salinity, while HPAM concentration is maintained constant at 1500 ppm. The contour plot shows a consistent decline in recovery factor as either temperature or salinity increases. Recovery values decrease from approximately 14–15% at low temperature and low salinity to approximately 8–9% at high temperature and high salinity. The response surface appears as a downward-sloping plane, indicating that both variables negatively influence polymer flooding performance. At a constant salinity of 10,000 ppm, increasing temperature from 20 °C to 80 °C reduces recovery by approximately 3 percentage points. Similarly, at a constant temperature of 20 °C, increasing salinity from 10,000 ppm to 100,000 ppm decreases recovery by roughly 4 percentage points. The simultaneous presence of high temperature and high salinity creates particularly unfavorable conditions for HPAM flooding. Temperature reduces molecular stability and viscosity retention, while salinity compresses polymer coils and decreases hydrodynamic volume. When both effects occur simultaneously, the polymer loses much of its mobility-control capability [73,74].

Figure 9 presents the interaction between salinity and HPAM concentration while temperature is maintained constant at 50 °C. This figure is particularly important because it compares the strongest positive factor (HPAM concentration) against one of the strongest negative factors (salinity). The contour plot demonstrates a pronounced increase in recovery factor with increasing polymer concentration and a simultaneous decrease with increasing salinity. Recovery values range from approximately 6–7% at low concentration and high salinity to nearly 17% at high concentration and low salinity. The response surface shows a steep upward trend along the concentration axis and a downward trend along the salinity axis. This indicates that concentration exerts a stronger influence on recovery than salinity, consistent with the trends observed in Figure 6. At a salinity of 10,000 ppm, increasing concentration from 500 ppm to 2500 ppm raises recovery from approximately 9% to about 17%, representing an increase of nearly 90%. In contrast, at a concentration of 1500 ppm, increasing salinity from 10,000 ppm to 100,000 ppm decreases recovery from approximately 13% to around 9–10%. The interaction originates from the dependence of polymer viscosity on ionic strength. High polymer concentrations increase chain entanglement and viscosity, whereas high salinity reduces chain expansion through electrostatic shielding. Consequently, salinity partially suppresses the beneficial effect of concentration [75]. Nevertheless, the surface plot demonstrates that increasing polymer concentration remains effective even under relatively saline conditions. From an operational perspective, Figure 9 indicates that polymer concentration can be used as a practical control parameter to compensate, at least partially, for adverse salinity conditions.

### 3.5. Optimization

Optimization was performed using the developed RSM-based recovery factor model to determine operating conditions that satisfy specific performance objectives. Two optimization scenarios were considered in this study, and the corresponding optimization criteria and results are summarized in Table 9.

**Figure 10:**
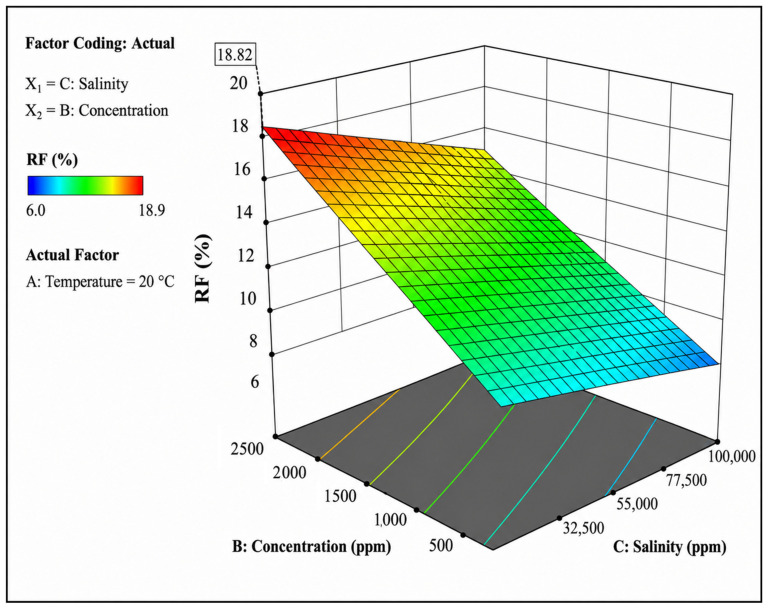
Response surface optimization plot showing the optimum operating conditions (20 °C, 2500 ppm HPAM, and 10,000 ppm salinity) for maximum RF, identified by the flag marker.

The first optimization objective (Goal I) aimed to maximize the recovery factor within the investigated design space. The optimization results predicted a maximum RF of 18.82%, achieved at a temperature of 20 °C, an HPAM concentration of 2500 ppm, and a salinity of 10,000 ppm. Figure 10 presents the response surface optimization plot generated at the optimum temperature of 20 °C. The optimum operating point is identified by the flag marker located on the response surface, corresponding to the highest predicted recovery factor within the investigated domain. This point represents the global optimum obtained from the developed RSM model and defines the most favorable combination of operating parameters for maximizing oil recovery.

**Figure 11:**
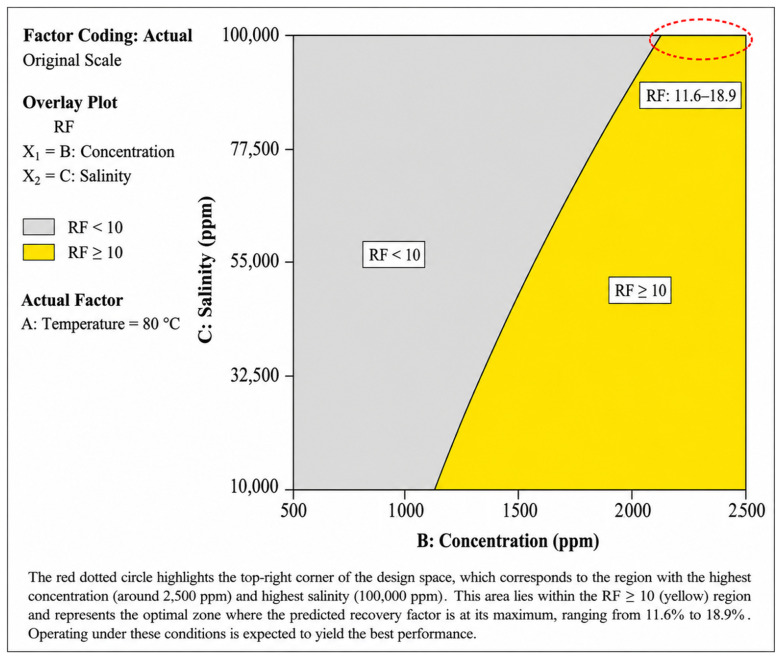
Optimization map at 80 °C and 100,000 ppm salinity showing the feasible region (RF > 10%) and the RF = 10 boundary line.

The second optimization objective (Goal II) was developed to identify operating conditions capable of maintaining a recovery factor greater than 10% under severe reservoir conditions. For this purpose, the temperature and salinity were fixed at their most unfavorable investigated values, namely 80 °C and 100,000 ppm, respectively, while HPAM concentration was allowed to vary. Figure 11 shows the optimization map generated under these conditions. The yellow region represents the feasible operating domain where RF exceeds 10%, whereas the gray region corresponds to conditions resulting in RF values below the target criterion. The boundary line separating the two regions represents the limiting condition of RF = 10%. According to the optimization results, a minimum HPAM concentration of approximately 2150 ppm is required to satisfy the target recovery factor under these harsh conditions. Increasing the HPAM concentration from 2150 to 2500 ppm resulted in RF values ranging from approximately 10% to 11%. Therefore, the optimization map provides a practical guideline for selecting polymer concentrations capable of maintaining acceptable flooding performance in reservoirs characterized by high temperature and high salinity.

Figure 11 is constructed based on the RSM-predicted recovery factor under fixed reservoir conditions of 80 °C temperature and 100,000 ppm salinity, while the HPAM concentration was varied within the studied range. The color map represents the predicted RF values obtained from the developed response surface model. The yellow region indicates the operating domain where the RF exceeds the target threshold of 10%, representing feasible and acceptable polymer flooding conditions. In contrast, the gray region corresponds to RF values below 10%, indicating non-feasible operating conditions under the imposed constraint. The separating boundary line between the two regions represents the iso-contour of RF = 10%, which defines the minimum HPAM concentration required to achieve the target recovery performance under severe reservoir conditions.

## 4. Conclusions

The present study investigated the effects of temperature, HPAM concentration, and salinity on HPAM flooding performance in matrix-type carbonate cores through laboratory core flooding experiments integrated with RSM and ANOVA. The incremental recovery factor varied from approximately 6.0% to 18.9%, confirming the strong dependence of polymer flooding efficiency on operating conditions. The developed quadratic model showed excellent predictive capability (R^2^ = 0.9991, *p* < 0.0001) and successfully captured the individual and interaction effects of the investigated variables.

Among the studied parameters, HPAM concentration exhibited the greatest influence on recovery factor, followed by salinity and temperature. Increasing polymer concentration from 500 to 2500 ppm enhanced recovery by approximately 80–100%, whereas increasing temperature from 20 to 80 °C and salinity from 10,000 to 100,000 ppm reduced recovery by about 20–25% and 25–35%, respectively. Response surface analysis further demonstrated significant interactions between HPAM concentration and salinity.

Optimization of the developed model predicted a maximum recovery factor of 18.82% at 20 °C, 2500 ppm HPAM concentration, and 10,000 ppm salinity. Under harsh reservoir conditions (80 °C and 100,000 ppm salinity), a minimum HPAM concentration of approximately 2150 ppm was required to maintain recovery factors above 10%. The novelty of this study lies in the integration of laboratory-scale HPAM flooding experiments with RSM–ANOVA modeling to establish a reliable predictive and optimization framework, providing practical guidance for polymer flooding design and implementation in carbonate reservoirs.

## Figures and Tables

**Table 1:** Summary of previous studies on polymer flooding optimization using RSM and ANOVA.

Authors	Flooding System	Methodology	Main Variables	Major Findings
Nguyen et al. [51]	Polymer flooding	D-optimal design, RSM	Polymer concentration, slug size	Optimum concentration of 1780 ppm identified; statistical design reduced simulation efforts
Douarche et al. [52]	Surfactant-polymer flooding	RSM, sensitivity analysis	Design and uncertain parameters	RSM significantly reduced computational cost and enabled process optimization
Hu and Li [53]	Polymer flooding	RSM, ANOVA	Injection rate, formation rhythm, injection timing	Injection rate was the dominant factor affecting incremental recovery
Liang et al. [45]	Heavy-oil polymer flooding	RSM	Polymer viscosity, residual resistance factor	Low-viscosity polymers with high RRF improved recovery while maintaining injectivity
Dabiri and Karaei [54]	HMPAM flooding	CCD, RSM, ANOVA	Pressure, salinity, polymer concentration	Polymer concentration had the greatest effect on IFT; optimized flooding increased recovery from 50.6% to 73.3%
Present study	HPAM flooding in carbonate cores	RSM, ANOVA, laboratory core flooding	Temperature, salinity, HPAM concentration	Developed predictive RF model, optimized flooding conditions, and identified minimum HPAM concentration required under harsh reservoir conditions

**Table 2:** Ionic composition of synthetic formation waters used in polymer flooding experiments.

Ion	High Salinity Brine	Medium Salinity Brine	Low Salinity Brine
Na^+^	28,218	15,075	1359
Ca^2+^	4000	4000	4000
K^+^	876	876	876
Mg^2+^	613	613	613
Cl^−^	66,341	34,402	3085
SO_4_^2−^	96	96	96
TDS	100,144	55,062	10,029

**Table 3:** Viscosity of solutions at different polymer concentrations measured at 25 °C and 55,000 ppm salinity.

HPAM Concentration (ppm)	Viscosity (cP)
500	4.5
1000	8.5
1500	13.5
2000	18.5
2500	24.0

**Table 4:** Viscosity of solutions at different temperatures for a polymer concentration of 1500 ppm and salinity of 55,000 ppm.

Temperature (°C)	Viscosity (cP)
20	13.5
40	11.2
60	9.0
80	7.2

**Table 5:** The experimental levels of the analyzed parameters.

Parameter	Symbol	Unit	Experimental Levels (Coded and Actual)		
			−1	0	+1
Temperature	A	°C	20	50	80
Concentration	B	ppm	500	1500	2500
Salinity	C	ppm	10,000	55,000	100,000

**Table 6:** DOE-RSM experimental design and results.

Run	Parameters (Coded)			Response
	A: Temperature	B: Concentration	C: Salinity	RF
	°C	ppm	ppm	%
1	0.000	−0.500	1.000	7.9
2	0.000	1.000	1.000	12.8
3	0.000	−1.000	0.000	7.6
4	−1.000	0.000	−1.000	14.7
5	−1.000	−1.000	−1.000	10.2
6	−1.000	−0.500	0.000	10.8
7	−1.000	−0.500	−1.000	12.4
8	0.000	0.000	0.000	11.2
9	0.000	−1.000	−1.000	9
10	0.000	0.500	1.000	11.1
11	1.000	0.000	0.000	9.7
12	−1.000	0.000	1.000	10.9
13	0.000	0.000	−1.000	13
14	0.000	1.000	−1.000	17.1
15	0.000	1.000	0.000	15
16	1.000	0.500	−1.000	13.2
17	1.000	1.000	1.000	11
18	−1.000	1.000	−1.000	18.9
19	1.000	0.000	1.000	8.1
20	1.000	−0.500	−1.000	9.5
21	−1.000	0.000	0.000	12.8
22	−1.000	0.500	1.000	12.4
23	1.000	1.000	−1.000	15.1
24	1.000	1.000	0.000	13.2
25	0.000	−1.000	1.000	6.1
26	0.000	0.500	0.000	13.1
27	−1.000	1.000	0.000	16.6
28	−1.000	−1.000	0.000	8.8
29	−1.000	−0.500	1.000	9
30	−1.000	0.500	0.000	14.5
31	0.000	−0.500	−1.000	11
32	−1.000	−1.000	1.000	7.1
33	1.000	0.500	1.000	9.6
34	1.000	−0.500	1.000	6.8
35	0.000	0.000	1.000	9.5
36	1.000	0.000	−1.000	11.3
37	0.000	−0.500	0.000	9.4
38	1.000	−0.500	0.000	8.2
39	1.000	0.500	0.000	11.4
40	0.000	0.500	−1.000	15
41	1.000	−1.000	1.000	6
42	−1.000	0.500	−1.000	16.5
43	−1.000	1.000	1.000	14.3
44	1.000	−1.000	0.000	6.7
45	1.000	−1.000	−1.000	7.8

**Table 7:** ANOVA results for the RF model developed by RSM.

Source	Sum of Squares	df	Mean Square	F-Value	*p*-Value	
Model	21,828.12	8	2728.51	5229.02	<0.0001	significant
A-Temperature	2925.05	1	2925.05	5605.67	<0.0001	
B-Concentration	13,686.29	1	13,686.29	26,228.87	<0.0001	
C-Salinity	4395.53	1	4395.53	8423.74	<0.0001	
AB	274.18	1	274.18	525.45	<0.0001	
AC	84.59	1	84.59	162.12	<0.0001	
BC	396.51	1	396.51	759.89	<0.0001	
B^2^	64.52	1	64.52	123.65	<0.0001	
C^2^	1.43	1	1.43	2.75	0.1060	
Residual	18.78	36	0.5218			
Cor Total	21,846.90	44				

**Table 8:** Fit statistics for the developed RF model.

No	Fit Statistics		RF Model
1	Coefficient of determination	R^2^	0.9991
2	Adjusted coefficient of determination	Adj-R^2^	0.9989
3	Predicted coefficient of determination	Pred-R^2^	0.9986
4	Adequate precision	A.P	288.7618
5	Coefficient of variation	C.V. %	1.45

**Table 9:** The optimization goals and corresponding results using the developed RF model.

Parameters	Unit	Goals		Optimization Results	
		I	II	I	II
Temperature	°C	in range	80	20	80
HPAM concentration	ppm	in range	in range	2500	2150–2500
Salinity	ppm	in range	100,000	10,000	100,000
RF	%	Maximize	>10	18.82	10–11

## Data Availability

The data used is provided in the article and can be used.
